# New reporter gene assays for detecting natural and synthetic molting hormone agonists using yeasts expressing ecdysone receptors of various insects

**DOI:** 10.1002/2211-5463.12239

**Published:** 2017-06-05

**Authors:** Sayoko Ito‐Harashima, Mai Matsuura, Masanobu Kawanishi, Yoshiaki Nakagawa, Takashi Yagi

**Affiliations:** ^1^ Department of BiologyGraduate School of ScienceOsaka Prefecture UniversitySakaiOsakaJapan; ^2^ Division of Applied Life SciencesGraduate School of AgricultureKyoto UniversitySakyo‐kuKyotoJapan; ^3^ Department of Life ScienceDongguk University Biomedical CampusGoyangGyeonggi‐doSouth Korea

**Keywords:** 20‐hydroxyecdysone, ecdysone receptor, insect growth regulator, molting hormone agonist, yeast reporter gene assay

## Abstract

Synthetic nonsteroidal ecdysone agonists, a class of insect growth regulators (IGRs), target the ecdysone receptor (EcR), which forms a heterodimer with ultraspiracle (USP) to transactivate ecdysone response genes. These compounds have high binding affinities to the EcR–USP complexes of certain insects and their toxicity is selective for certain taxonomic orders. In the present study, we developed reporter gene assay (RGA) systems to detect molting hormone (ecdysone) activity by introducing EcR–USP cDNA and a bacterial *lacZ* reporter gene into yeast. EcR and USP were derived from the insect species of three different taxonomic orders: *Drosophila melanogaster* (Diptera), *Chilo suppressalis* (Lepidoptera), and *Leptinotarsa decemlineata* (Coleoptera). Transcriptional coactivator taiman (Tai) cDNA cloned from *D. melanogaster* was also used in this RGA system. This yeast RGA system responded to various EcR ligands in a dose‐dependent and ecdysteroid‐specific manner. Furthermore, the insect order‐selective ligand activities of synthetic nonsteroidal ecdysone agonists were linearly related to their binding activities, which were measured against *in vitro* translated EcR–USP complexes. Our newly established yeast RGA is useful for screening new molting hormone agonists that work selectively on target insects.

**Abbreviations**
20E: 20‐hydroxyecdysone
AhR: aryl hydrocarbon receptor
DBH: dibenzoylhydrazine
DMSO: dimethyl sulfoxide
DR: direct repeat
DTT: dithiothreitol
E2: 17β‐estradiol
EcR: ecdysone receptor
EcRE: ecdysone response element
E: Ecdysone
ER: estrogen receptor
ER: everted repeat
GRE: glucocorticoid‐responsive element
GR: glucocorticoid receptor
IGD: insect growth disruptor
IGR: insect growth regulator
IR: inverted repeat
JH: juvenile hormone
LBD: ligand‐binding domain
MH: molting hormone
MR: mineralocorticoid receptor
OD: optical density
ONPG: *o‐*nitrophenyl‐β‐d‐galactopyranoside
Pon A: ponasterone A
RAR: retinoid acid receptor
RE: responsive elements
RXR: retinoid X receptor
Tai: taiman
THQ: tetrahydroquinoline
TR: thyroid hormone receptor
USP: ultraspiracle

Insect development is regulated by two types of insect‐specific peripheral hormones: molting hormones (MHs) and juvenile hormones (JHs). 20‐Hydroxyecdysone (20E) and JH‐III are commonly used as MHs and JHs, respectively, in most insects. Compounds that mimic these hormones may be used as insecticides, which are categorized as insect growth regulators (IGRs) or insect growth disruptors (IGDs) 1. Five MH and two JH agonists are used as insecticides in the agricultural market 2.

The activities of MHs and JHs were previously measured using a simple traditional bioassay system that required the whole body such as ligation, injection, topical, and dip methods (*in vivo*) 3, 4 prior to the development of molecular techniques. *In vitro* systems using imaginal disks 5 and cultured integuments 6, 7 have since been developed in order to measure the activities of insect hormones and IGRs. The cultured integument system used to detect molting hormone activity was later modified by Nakagawa *et al*. 8, 9, in which the induction of chitin synthesis by 20E was measured. Intact insect cells or *in vitro* translated receptor proteins have recently been used to measure ligand–receptor binding affinity 10, 11, 12, 13.

The chemical structures of MHs were identified in 1964 14, 15, and the ecdysone receptor (EcR) and its partner protein ultraspiracle (USP) were identified in the fruit fly *Drosophila melanogaster* using cDNA cloning techniques 16, 17, 18. EcR and USP were found to be homologous to vertebrate nuclear receptor proteins, and EcR–USP complexes derived from various insect species have been characterized 19. Ligand–receptor binding was elucidated using a crystal structure analysis 20, which accelerated the *in silico* ligand design 21. Under these conditions, various MH‐like compounds with novel chemical structures have been discovered and some have been chemically synthesized 2. The identification of EcR and USP genes has resulted in *in vitro* ligand–receptor‐binding assays 12, 22, 23.

Reporter gene assay is also used as an alternative method to detect the MH activities of artificial compounds. EcR–USP‐dependent RGA is based on the quantitation of the expression of reporter genes in various insect cells 23, 24, 25, 26, 27, 28; however, MH activity evaluated using this assay varies due to the presence of endogenous species‐specific EcR–USP and cofactors. If it is possible to reconstitute the RGA system in EcR–USP‐free cells such as yeasts, this assay may have the capacity to measure MH activity directly by expressing the EcR–USP of various insect species. We previously established yeast RGA to detect environmental contaminants containing vertebrate nuclear receptor ligands 29, 30, 31, 32, 33, 34, 35, 36, 37. Yeast RGAs are simpler, easier to handle, and less expensive than mammalian cell‐based bioassays and instrumental analyses.

In the present study, we newly developed yeast RGAs to quantitatively measure the activities of MHs in recombinant yeast strains. EcR and USP derived from three different insect species: the dipteran *D. melanogaster*, lepidopteran *Chilo suppressalis*, and coleopteran *Leptinotarsa decemlineata*, were expressed along with the transcriptional coactivator taiman (Tai) from *D. melanogaster* (Fig. 1). Tai is one of key components to construct the yeast RGAs since it binds to EcR–USP in a ligand‐dependent manner and potentiates transcriptional activation in insect cultured cells 38. We compared the responses of EcR–USP against natural steroid hormones among the three insect species using established EcR–USP assay yeasts. We also examined whether this yeast RGA has the ability to measure the insect‐selective effects of synthetic nonsteroidal ecdysone agonists.

**Figure 1:**
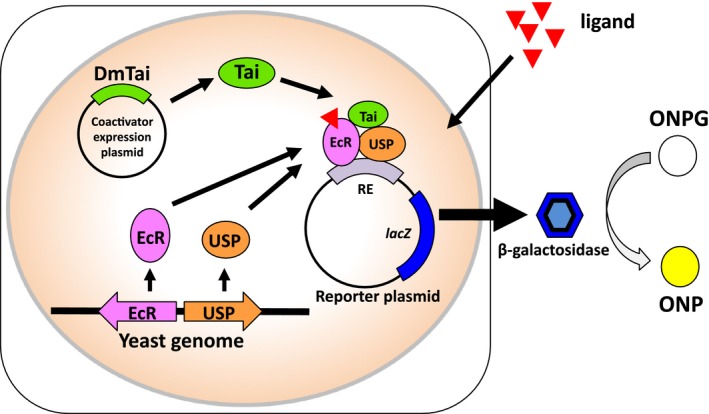
Principle of the RGA with yeast strains expressing insect ecdysone receptors. The ecdysone receptors EcR and USP expressed in yeast cells bind to upstream response elements of the *lacZ* reporter gene in response to ligands. Tai, a transcriptional coactivator, cooperates with EcR–USP and enhances the induction of β‐galactosidase. The expression of β‐gal may be visualized and quantified by the development of a yellow color due to the accumulation of ONP in the assay buffer.

## Materials and methods

### Strains and media

The *Escherichia coli* strain, DH5α, was used as a host strain to amplify plasmid DNA. *Saccharomyces cerevisiae* W303a (*MAT*a, *ura3‐1*,* ade2‐1*,* trp1‐1*,* leu2‐3*,* his3‐11*,* 15*,* can1‐100*) was used to establish reporter assay yeast strains. Yeast extract peptone dextrose (YPD) and synthetic dextrose complete dropout (SDC‐X) media were prepared as previously described 39. Synthetic galactose complete dropout (SGC‐X) media contained 2% (w/v) galactose instead of dextrose. All solid media contained 2% (w/v) agar in plates.

### Chemicals

A tetrahydroquinoline (THQ)‐type compound (C_23_H_19_BrF_2_N_2_O) was synthesized as previously described 40, 41. Dimethyl sulfoxide (DMSO), tebufenozide, methoxyfenozide, chromafenozide, corticosterone, hydrocortisone, and testosterone were purchased from Wako Pure Chemical Industries, Ltd. (Osaka, Japan). Dithiothreitol (DTT), 17β‐estradiol (E2), and progesterone were obtained from Nacalai Tesque (Kyoto, Japan). 20‐Hydroxyecdysone (20E), ecdysone, ponasterone A (Pon A), aldosterone, halofenozide, and *o‐*nitrophenyl‐β‐d‐galactopyranoside (ONPG) were purchased from Sigma Aldrich Chemical Co. (St. Louis, MO, USA). Restriction enzymes, DNA modification enzymes, and other chemicals were obtained from Wako Pure Chemical Industries, Ltd., TaKaRa Bio Inc. (Otsu, Japan), or TOYOBO Co., Ltd. (Osaka, Japan).

### Plasmid construction

The EcR–USP and DmTai expression plasmids and the reporter plasmid carrying the ecdysone response element (EcRE) from the *D. melanogaster hsp27* gene were constructed for the development of EcR–USP ligand reporter assays. The primer sequences used in this study were synthesized by Sigma‐Aldrich (Tokyo, Japan) and are listed in Table S1.

**Figure 2:**
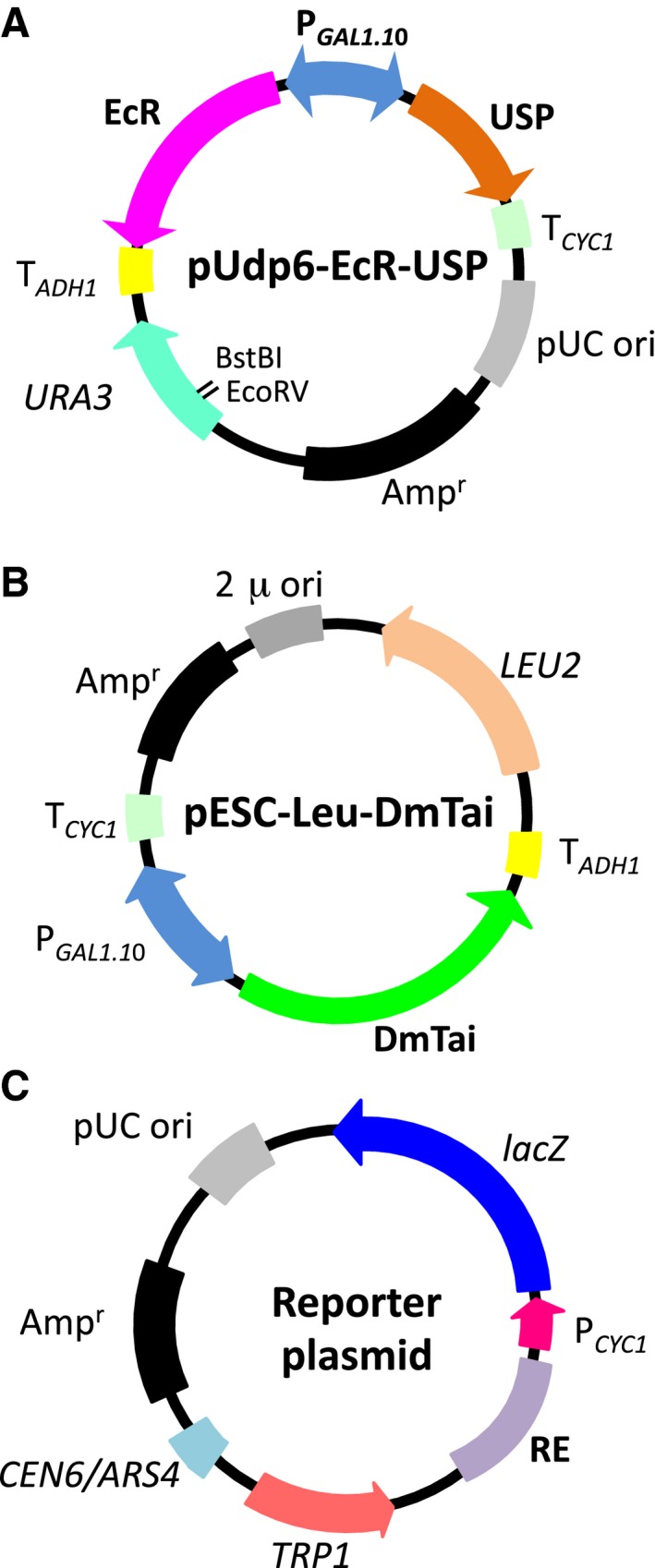
Maps of plasmids used in this study. Plasmid maps of the EcR‐USP expression plasmids pUdp6‐EcR‐USP (A), coactivator expression plasmid pESC‐Leu‐DmTai (B), and reporter plasmids (C). In the map, P_*GAL*__*1, 10*_: *GAL1, 10* dual directional promoter; T_*ADH*__*1*_: terminator sequence of yeast alcohol dehydrogenase gene 1 (*ADH1*); T_*CYC*__*1*_: terminator sequence of yeast cytochrome C gene 1 (*CYC1*); the *Eco*RV and *Bst*BI sites were indicated in (A).

The DNA fragments containing the DmEcRB1 (GenBank accession number M74078) and DmUSP (NM_001272239) ORFs were obtained using a PCR from the plasmids, pCMA‐EcR‐B1and pCMA‐USP 42, with the primer pairs DmEcRFwd and DmEcR Rev, and DmUSP Fwd and DmUSP Rev, respectively, which contain a restriction site and/or yeast ribosomal binding consensus sequence near the initiation codon. PCR was performed with high‐fidelity PCR polymerase KOD‐plus‐ver. 2 or KOD‐plus‐Neo (TOYOBO Co. Ltd.) according to the manufacturer's instructions. The amplified fragments were digested with *Sal*I and *Hin*dIII for DmEcR, and *Sma*I and *Eco*RI for DmUSP, and cloned into the corresponding sites of multicloning site (MCS) 1 and MCS2 of the expression vector pUdp6 33, respectively. Similarly, CsEcRB1 (AB067812) and CsUSP (AB081840) were amplified from the plasmids pEU3‐NII‐EcR and pEU3‐NII‐USP, respectively 43. LdEcRA (AB211191) and LdUSP (AB211193) ORFs were derived from pET‐23a(+)‐LdEcR and pET‐23a(+)‐USP 22, respectively. PCR fragments were digested with *Bam*HI and *Hin*dIII for CsEcR, *Sma*I and *Eco*RI for CsUSP, *Xba*I and *Hin*dIII for LdEcR, and *Sma*I and *Sac*I for LdUSP, and inserted into the corresponding sites of pUdp6. The resultant plasmids were designated as pUdp6‐DmEcR, pUdp6‐DmUSP, pUdp6‐CsEcR, pUdp6‐CsUSP, pUdp6‐LdEcR, and pUdp6‐LdUSP, respectively. The plasmids were isolated and purified using a QIAGEN Mini Prep Kit (Valencia, CA, USA), and the nucleotide sequences of EcR and USP ORFs were confirmed using the ABI DNA sequencer. In order to construct EcR–USP heterodimer‐expressing plasmids, the DNA fragment containing the USP gene was excised from each USP‐expressing plasmid, and cloned into the MCS2 of each EcR‐expressing plasmid of the corresponding insect. The plasmids obtained were designated as pUdp6‐DmEcR‐USP, pUdp6‐CsEcR‐USP, and pUdp6‐LdEcR‐USP, respectively (Fig. 2A).

The ORF of the transcriptional coactivator DmTai was amplified from *D. melanogaster* larva poly A^+^ RNA (Clontech, Palo Alto, CA, USA) by a RT‐PCR. cDNA was obtained using SuperScript III reverse transcriptase (Invitrogen, Carlsbad, CA, USA). DmTai cDNA was amplified by PCR using KOD‐plus Neo DNA polymerase with the primers DmTai Fwd and DmTai Rev, which were designed based on the nucleotide sequence registered in the DDBJ/EMBL/GenBank database (ID: AY008258). An amplified DNA fragment of ~ 6 kb was cloned into the pCR2.1 TOPO vector (Invitrogen) by TA cloning and sequenced. The cloned DNA fragment containing DmTai ORF was excised out from the pCR2.1 TOPO vector by digestion with *Bgl*II and *Hin*dIII, and inserted into the *Bam*HI–*Hin*dIII sites of multicloning site (MCS) 2 on the yeast expression vector pESC‐Leu (Agilent Technologies, Inc., Santa Clara, CA, USA). We found significant numbers of sequence alterations including four in‐frame insertions/deletions and three amino acid substitutions in cloned DmTai ORF, which were distinct from the sequence of AY008258. The most notable difference between our DmTai clone and AY008258 was an in‐frame deletion of 63 bp. A comparison of the nucleotide and deduced amino acid sequences with those of the DmTai variants C, D, E, F, G, and H registered in the DDBJ/EMBL/GenBank database revealed that our DmTai clone was amplified from variant C (NM_001201817) or D (NM_078797) mRNAs. Since this plasmid exhibited functional transcriptional coactivation activity against EcR–USP in yeast cells, we designated this plasmid as pESC‐Leu‐DmTai (Fig. 2B) and used it to establish yeast strains for EcR–USP assays in this study. The expression of EcR–USP and DmTai in yeast is under the control of the galactose‐driven *GAL1, 10* dual directional promoter.

In order to construct the reporter plasmids pYTβ‐Dmhsp27 × 1, pYTβ‐Dmhsp27 × 2, and pYTβ‐Dmhsp27 × 3 carrying the ecdysone‐responsive element (EcRE) found on the promoter region of *D. melanogaster hsp27*, one to three copies of *hsp27* EcRE‐containing oligonucleotides were inserted into the pRW95‐3 vector 44 upstream of the CYC1 minimal promoter. The oligonucleotides Dmhsp27 Fwd and Dmhsp27 Rev (TableS1) were annealed, phosphorylated with T4 polynucleotide kinase, ligated, and inserted into the *Spe*I site of pRW95‐3. The copy numbers and orientation of Dmhsp EcRE on each plasmid were confirmed by sequencing. Other reporter plasmids carrying responsive elements (RE) for human nuclear receptors [Inverted repeat (IR)‐, direct repeat (DR)‐, everted repeat (ER)‐type REs, and glucocorticoid‐responsive element (GRE)] that had been previously constructed in our laboratory 33, 35, 36 (Fig. 2C and TableS2) were also used to optimize the reporter assay for EcR–USP assays. pYTβ‐IR0 × 5, pYTβ‐DR2 × 4, and pYTβ‐DR4 × 7 contained five copies of IR0 (AGGTCATGACCT), four copies of DR2 (AGCGGATAAGGTCA), and seven copies of DR4 (AGGTCACAGGAGGTCA), respectively, were chosen as reporter plasmids to establish yeast strains for the DmEcR‐USP, CsEcR‐USP, and LdEcR‐USP assays, respectively.

### Establishment of the yeast RGA

Yeast transformation was performed using the lithium acetate procedure as previously described 45. In this yeast RGA method, one of the reporter plasmids and the expression plasmid for DmTai, pESC‐Leu‐DmTai, were introduced into the wild‐type yeast strain W303a. A transformant grown on SDC‐TRP/LEU agar medium was isolated and used as a host for subsequent transformation. The EcR–USP expression plasmid pUdp6‐DmEcR‐USP was linearized by *Eco*RV digestion, and pUdp6‐CsEcR‐USP and pUdp6‐LdEcR‐USP were linearized by *Bst*BI digestion and integrated into the *ura3* locus in the yeast genome by homologous recombination. Transformants were selected on SCD‐TRP/LEU/URA agar plates. Plasmids expressing EcR or USP only were also linearized by *Eco*RV (DmEcR and DmUSP) or *Bst*BI (CsEcR, CsUSP, LdEcR, and LdUSP) digestion, and introduced into W303a, as described above.

### Measurement of MH activity using the yeast RGA

This RGA was conducted as described previously 36. Single colonies of the yeast strains were grown in SDC‐TRP/LEU/URA medium at 30 °C overnight, and the optical density (OD) at 595 nm of each culture was adjusted to 1.0 with the same medium. A 1‐μL aliquot of the test chemicals dissolved in DMSO, 10 μL of the overnight culture yeast, and 90 μL of SGC‐TRP/LEU/URA (to induce EcR–USP and DmTai expression) were mixed in a 96‐well polystyrene microplate with subsequent incubation for 18 h at 30 °C. Each cell suspension (10 μL) was transferred to a new 96‐well microplate and 100 μL of Z‐buffer (60 mm Na_2_HPO_4_, 40 mm NaH_2_PO_4_, 1 mm MgSO_4_, 10 mm KCl, 2 mm DTT, and 0.2% sarcosyl, adjusted to pH 7.0) containing 1 mg·mL^−1^ ONPG was added to the plates with subsequent incubation at 37 °C for 60 min. Absorbance at wavelengths of 405 and 595 nm was measured using Micro Plate Reader Model 680 (BioRad Laboratories, Inc., Hercules, CA, USA) in order to estimate β‐galactosidase activity as the amount of *o*‐nitrophenol produced and yeast cell density, respectively. Agonist‐dependent *lacZ* reporter induction was demonstrated as ‘an increase in induction’, which was calculated using the following formula: [OD_405_ (sample)/OD_595_ (sample)] − [OD_405_ (DMSO)/OD_595_ (DMSO)]. Data were analyzed using the Student's *t*‐test to assess significance between two sets of values. Probability (*P*) values < 0.01 were considered significant. Based on the dose–response curves of the reporter assays in each strain, the 50% effective concentration [EC_50_ (μm)] for each compound was evaluated using Probit transformation 46.

## Results

### Optimization of the yeast RGA

In order to optimize RGA for the EcR–USP of three insect species from different taxonomic orders, we constructed new reporter plasmids carrying EcRE of the *Dmhsp27* promoter, which was first identified as EcRE 47. As shown in Fig.S1A, the yeast strains carrying DmEcR–USP exhibited reporter activities in response to 20E and increased transactivation in a manner that depended on the copy number of the Dmhsp27 EcRE motif. We also tested reporter plasmids carrying IR, DR, ER, and GRE as the response element. DmEcR‐USP strongly activated 20E‐induced reporter gene expression in yeast via ER6, IR0, IR4, DR1, DR4, and DR5 (Fig.S1B). IR0 was the most responsive element among these response elements. The reporter activity of the yeast strain carrying IR0 was markedly higher than that of the strain carrying three copies of Dmhsp27 EcRE (Fig.S1A,B). Therefore, we selected pYT‐IR0 × 5 containing five copies of IR0 as a reporter plasmid for the DmEcR‐USP assay in this study. We also investigated whether the coactivator DmTai exhibited the ability to enhance the responses of DmEcR‐USP. As shown in Fig.S1B, the expression of DmTai markedly enhanced the response against 20E in yeast strains expressing DmEcR‐USP.

Similarly, yeast strains expressing CsEcR‐USP and LdEcR‐USP along with DmTai were also established and responses to 20E were compared among various response elements. In CsEcR‐USP strains, a number of response elements functioned as EcRE (Fig.S1C). Among these response elements, DR2 was chosen for the EcRE of CsEcR‐USP because this strain showed less ligand‐independent β‐galactosidase activity than the others (data not shown). Regarding LdEcR‐USP, DR4 was the most efficient EcRE (Fig.S1D). We selected the yeast strains carrying pYT‐DR2 × 4 and pYT‐DR4 × 7 as reporter plasmids for CsEcR‐USP and LdEcR‐USP, respectively, and further investigated their responses to endogenous and synthetic EcR ligands.

**Figure 3:**
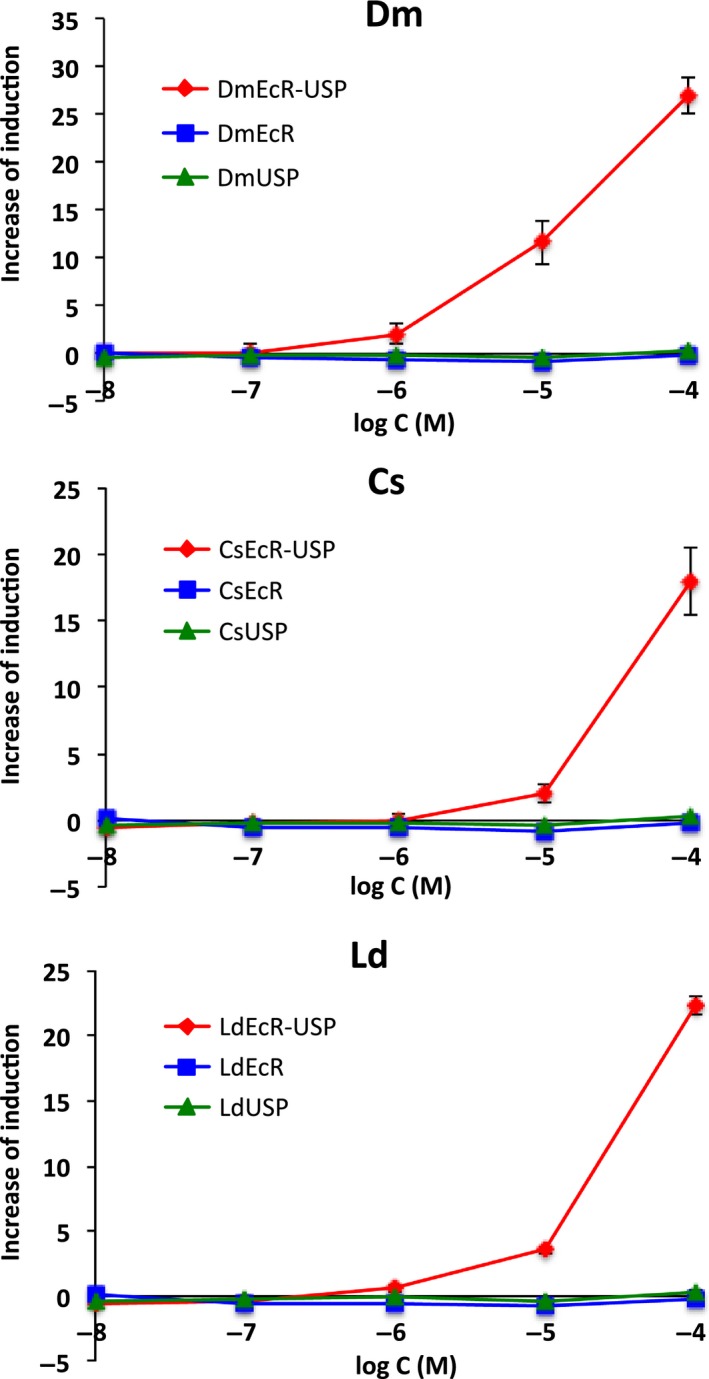
Requirement of USP expression for ligand responses in yeast strains expressing insect EcRs. The 20E responses of the yeast strains coexpressing EcR and USP (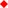) were compared with those expressing EcR (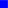) or USP (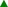) alone. Data represent the mean ± SD of triplicate experiments.

We also compared ligand responses in yeast strains expressing EcR–USP, EcR, and USP, respectively. Only the strains coexpressing EcR and USP induced *lacZ* reporter gene expression in response to 20E, while none of the strains expressing EcR or USP alone responded to 20E (Fig. 3).

### Responses of the yeast RGA to endogenous and plant‐derived ecdysteroids

**Figure 4:**
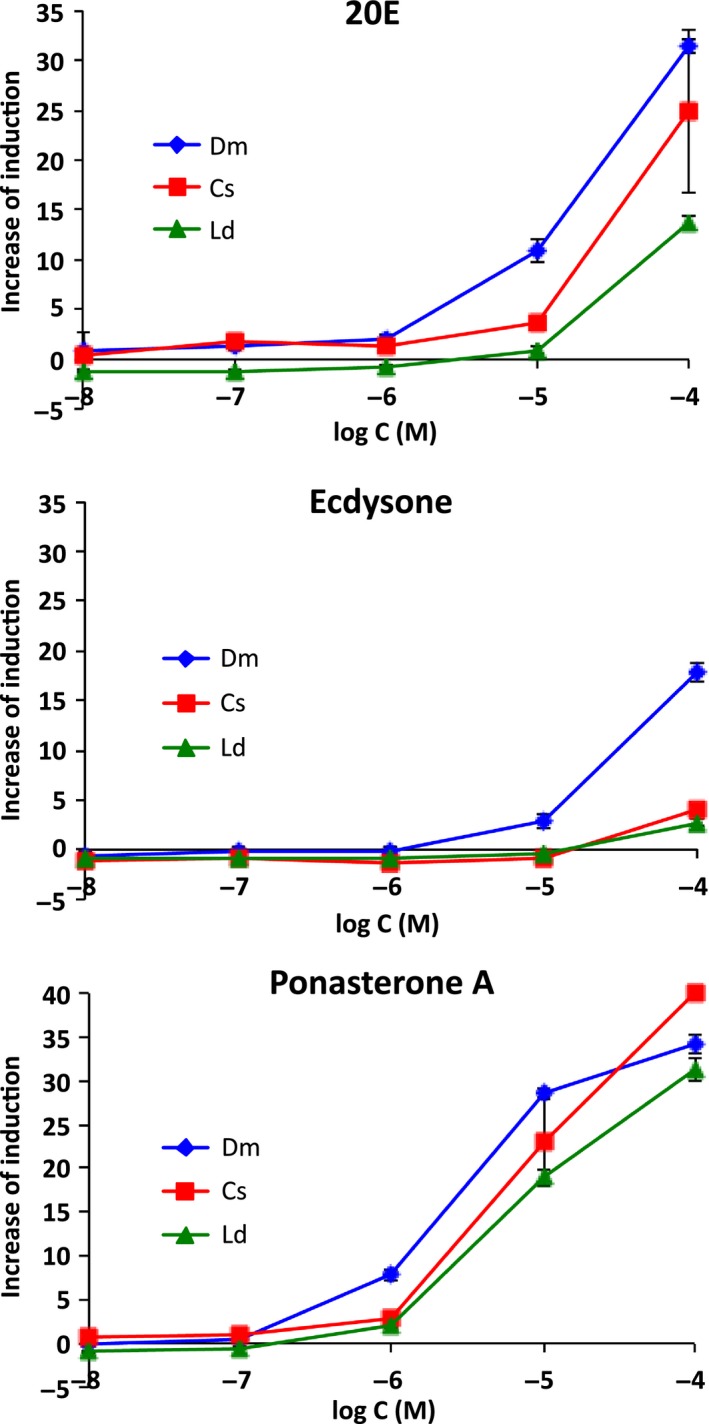
Responses to endogenous and plant‐derived ecdysteroids in yeast strains expressing DmEcR‐USP (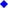), CsEcR‐USP (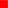), and LdEcR‐USP (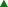). Yeast reporter assays were conducted using 20E, ecdysone, or ponasterone A as ligands. Data represent the mean ± SD of triplicate experiments.

**Figure 5:**
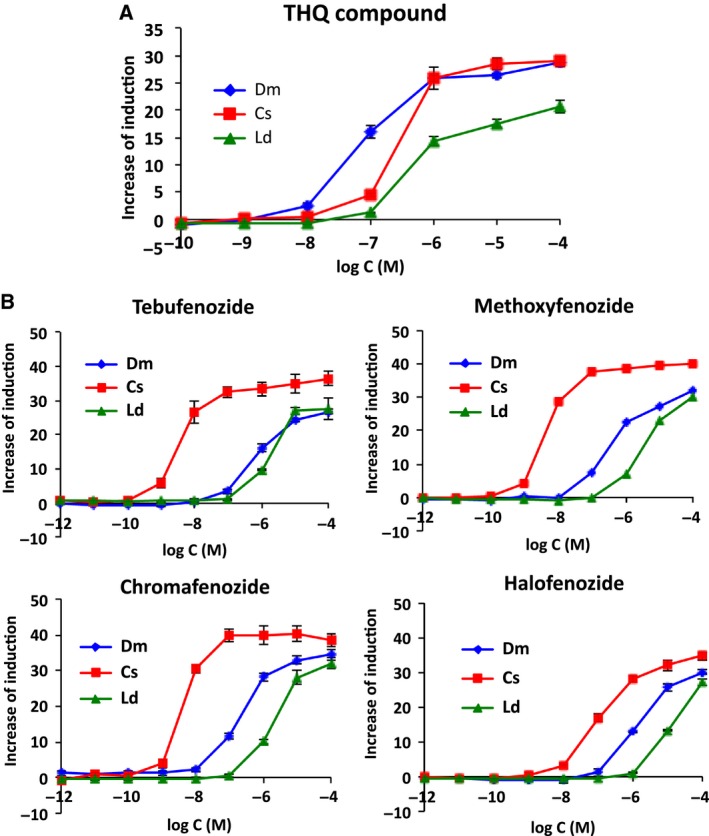
Responses of EcR–USP assay yeasts against synthetic ecdysteroid agonists. The yeast strains expressing DmEcR‐USP (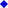), CsEcR‐USP (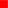), and LdEcR‐USP (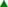) were exposed to THQ compound (A) and four DBHs: tebufenozide, methoxyfenozide, chromafenozide, and halofenozide (B), and the ligand‐dependent induction of β‐gal was measured. Data represent the mean ± SD of triplicate experiments.

As shown in Fig. 4, all yeast strains expressing EcR–USP responded to 20E and plant‐derived Pon A in dose‐dependent manners. Ecdysone (E), a precursor of 20E, slightly induced the *lacZ* reporter gene at high doses. The EC_50_ values of the ecdysteroids calculated from dose–response curves are shown in Table 1. Pon A was more potent than 20E in all EcR–USP assay yeast strains. The ligand potency of E was markedly less than that of 20E and Pon A. The order of potency was Pon A > 20E > E with all insect species (*P* < 0.01). Among the three species, DmEcR‐USP was more responsive to all these ligands than the other two EcR–USP. Significant differences were observed in EC_50_ values (*P* < 0.01) for 20E (Dm vs. Ld) and Pon A (Dm vs. Cs/Ld; Table 1). Our yeast RGA did not respond to vertebrate steroid hormones or alkylphenol compounds (Figs S2 and S3). Only ecdysteroids and synthetic ecdysone agonists induced reporter gene expression in this yeast RGA (Figs 4 and 5, see below).

**Table 1:** Detection limit and EC_50_ of endogenous and plant‐derived ecdysteroids in EcR–USP assay yeasts. n.d., not determined

	DmEcR‐USP		CsEcR‐USP		LdEcR‐USP	
	Detection limit (μm)	EC_50_ (μm)	Detection limit (μm)	EC_50_ (μm)	Detection limit (μm)	EC_50_ (μm)
20E	10	12 ± 0.39	10	15 ± 1.1	10	16 ± 0.28
Ecdysone	10	14 ± 0.77*	100	n.d.	100	n.d.
Pon A	1	2.8 ± 0.13**	1	6.5 ± 0.96**	1	6.2 ± 0.21**

EC_50_ values of *ecdysone and **pon A were significantly different (*P* < 0.01) from that of 20E in each yeast strain.

### Responses to synthetic nonsteroidal ecdysone agonists

A Diptera‐selective THQ compound and four Lepidoptera‐selective dibenzoylhydrazine (DBH) compounds 1, 40, 48 were submitted to the yeast RGA. RGAs with all EcR–USP responded to these ligands in a dose‐dependent manner (Fig. 5). In the assay with the THQ compound, the DmEcR‐USP assay yeast strain was the most responsive with a 10‐fold lower minimum detection limit than the other two EcR–USP‐expressing yeast strains. The EC_50_ value of THQ in the DmEcR‐USP assay yeast strain was approximately three‐ and eightfold lower than those in the CsEcR‐USP and LdEcR‐USP assay yeast strains, respectively (*P* < 0.01; Fig. 5A and Table 2). Insect‐selective responses were prominent in the assay of DBHs. The sensitivity of the CsEcR‐USP assay yeast strain was the highest among the three EcR–USP assay yeast strains. The CsEcR‐USP yeast strain responded to DBHs at 10^−9^ m, except for halofenozide, and reporter activities reached the maximum level at 10^−7^ m (Fig. 5B). Although halofenozide was less potent than the other three compounds in this yeast RGA, it was consistent with previous findings showing that its affinity with *in vitro* translated CsEcR‐USP was significantly lower than other DBHs 49. The concentrations of the minimum detection limits for tebufenozide, methoxyfenozide, and chromafenozide in the CsEcR‐USP assay yeast strain were 100‐ and 1000‐fold lower, and EC_50_ values were ~ 60–190‐fold and 450–630‐fold lower than those in the DmEcR‐USP and LdEcR‐USP assay yeast strains, respectively (*P* < 0.01). Although the insect‐selective activity of halofenozide was not as strong as the other three DBHs, the sensitivity of CsEcR‐USP was significant (*P* < 0.01): 10‐ and 100‐fold lower minimum detection limits, and ~ 8‐ and 50‐fold lower EC_50_ values than DmEcR‐USP and LdEcR‐USP, respectively (Fig. 5B and Table 2).

**Table 2:** Detection limit and EC_50_ of artificial ecdysteroid agonists in EcR–USP assay yeasts

	DmEcR‐USP		CsEcR‐USP		LdEcR‐USP	
	Detection limit (μm)	EC_50_ (μm)	Detection limit (μm)	EC_50_ (μm)	Detection limit (μm)	EC_50_ (μm)
THQ compound	0.01	0.11 ± 0.021	0.1	0.34 ± 0.028*	0.1	0.83 ± 0.062**
Tebufenozide	0.1	0.75 ± 0.16#	0.001	0.0040 ± 0.00048	1	2.3 ± 0.44##
Methoxyfenozide	0.1	0.55 ± 0.073#	0.001	0.0051 ± 0.00069	1	3.2 ± 0.39##
Chromafenozide	0.1	0.29 ± 0.016#	0.001	0.0049 ± 0.00029	1	2.2 ± 0.39##
Halofenozide	0.1	1.5 ± 0.33#	0.01	0.19 ± 0.022	1	9.3 ± 0.46##

In the assay of THQ compounds, EC_50_ values were significantly different (*P* < 0.01) between DmEcR‐USP and other insect species: *DmEcR‐USP vs. CsEcR‐USP; **DmEcR‐USP vs. LdEcR‐USP. In the assay of DBHs, EC_50_ values were significantly different between CsEcR‐USP and other insect species: ^#^CsEcR‐USP vs. DmEcR‐USP; ^##^CsEcR‐USP vs. LdEcR‐USP.

### Relationship between ligand potency in the yeast RGA and receptor‐binding activity

**Figure 6:**
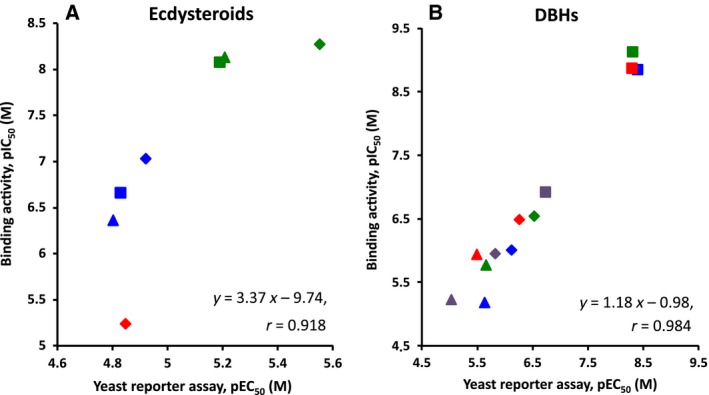
Relationships between ligand potency in yeast reporter assays (pEC_50_) and binding activities (pIC_50_) of various ecdysteroids as well as artificial nonsteroidal ecdysone agonists to DmEcR‐USP (diamond marks), CsEcR‐USP (square marks), and LdEcR‐USP (triangle marks). (A) Comparison of the endogenous ecdysteroids 20E (blue marks), ecdysone (red marks), and plant‐derived ponasterone A (green marks). (B) Comparison of the DBHs, tebufenozide (blue marks), methoxyfenozide (red marks), chromafenozide (green marks), and halofenozide (purple marks). Correlation coefficiencies (*r*) are shown in each graph. The pIC_50_ values referred as binding activity were from data reported by Minakuchi *et al*. 49.

Potency in terms of the pEC_50_ of natural ecdysteroids and the synthetic nonsteroidal ecdysone agonists evaluated in this yeast RGA was compared with receptor‐binding activity (pIC_50_) 49. In the yeast RGA, the potencies of 20E and pon A were 40–130‐fold and 520–840‐fold, respectively, lower than their binding activities against EcR–USP: the correlation coefficient was not high (*r* = 0.918), and the slope of the regression line and intercept were far from 1 and 0, respectively. On the other hand, differences between EC_50_ in the yeast RGA and IC_50_ for the receptor‐binding activities of DBHs were within 6.6‐fold at most. The correlation coefficient of the regression line for DBHs was high (*r* = 0.984), with the slope of the regression line and intercept being close to 1 and 0, respectively (Fig. 6), indicating that the ligand potencies of DBHs in the yeast RGA strongly correlated with their receptor‐binding activities.

## Discussion

Reporter gene assays are widely used to detect nuclear receptor ligands. We previously constructed RGAs using yeast cells that mimic cellular processes with intact nuclear receptors such as aryl hydrocarbon receptor (AhR), estrogen receptor (ER), glucocorticoid receptor (GR), mineralocorticoid receptor (MR), thyroid hormone receptor (TR), retinoid acid receptor (RAR), and retinoid X receptor (RXR) from vertebrates [29, 31, 32, 33, 35, 36, 37]. Insect EcRs and USPs belong to the nuclear receptor superfamily, and function as ligand‐dependent transcription factors. EcR–USP bind to EcRE and enhance the transcription of downstream genes with the binding of molting hormones 17, 18, 50, 51. To date, RGAs for insect EcR–USP have been developed in insect cells 23, 24, 25, 26, 27, 28. Mammalian cells do not have EcR–USP, and are considered to be suitable for the construction of RGAs for the EcR–USP ligand interaction system without perturbation by insect endogenous EcR–USP 52. However, mammals have RXRs that are USP homologs and have the ability to heterodimerize with EcR 17, 18, 53, 54. This may lead to mis‐estimations of ligand potency in mammalian cells. The yeast RGA expressing EcR–USP has the advantage of directly measuring MH activity because yeasts do not have nuclear receptors (Fig. 1).

In order to elucidate the regulatory mechanisms underlying EcR–USP and their ligand specificities in more detail, the reconstitution of transcriptional regulation was previously undertaken in yeasts. However, the ligand‐dependent transactivation activities of EcR–USP were not detected even though the ligand binding of EcR expressed in yeast cells was confirmed to be dependent on USP 55, 56, 57. We herein showed that DmTai is necessary for the ligand‐dependent transcriptional activation of EcR–USP in yeast (Fig.S1B). This is consistent with previous findings showing that the ligand‐dependent transactivation of EcR from *Choristoneura fumiferana* and *Aedes aegypti* was only detected in the presence of the mouse transcriptional coactivator GRIP1 in yeast 53, 54. DmTai is a *Drosophila* homolog of the vertebrate p160 family steroid hormone receptor coactivators [SRC‐1, 2 (mouse GRIP1), 3] 38, and may potentiate EcR–USP‐dependent transcriptional activation via histone acetylation and/or recruitment of the other transcriptional coactivators 58. We next showed that the response elements ER, IR, and DR motifs were more effective than Dmhsp27 in 20E‐dependent gene expression (Fig.S1). This indicates that the EcR–USP of these insects have the ability to bind to these response elements in addition to hsp27 59, 60, 61, 62, 63.

As shown in Fig. 3, 20E‐dependent gene expression was only observed in transgenic yeast strains with both EcR and USP genes, suggesting that the heterodimerization of EcR and USP is necessary for ligand‐dependent gene transactivation in yeasts. This result is consistent with previous findings showing that EcR require USP for ligand binding and binding to EcRE 12, 17, 18, 22, 64. The yeast strains did not respond to vertebrate steroid hormones, which are structurally similar to ecdysteroids and alkylphenols with endocrine‐disrupting activities against vertebrates and invertebrates including insects 33, 65, 66, 67, 68, 69 (Figs S2 and S3), indicating that our yeast RGA specifically responds to ligands with ecdysteroid activity.

20E and PonA both dose‐dependently induced *lacZ* reporter gene expression (Fig. 4). Ecdysone, which is a precursor of 20E and has weak MH activity in insects 70, also exhibited ligand ability in the yeast RGA (Fig. 4). The ligand responses observed in our yeast RGA are consistent with the ecdysteroid‐specific formation of phagocytotic clumps and transactivation activities of DmEcR‐USP in *Drosophila* cell lines 71, 72 as well as to the binding affinities of these compounds to *in vitro* translated EcR–USP 49.

Some synthetic nonsteroidal ecdysone agonists exhibit insecticidal activities by disrupting the normal molting process in a taxonomic order‐selective manner. The insect‐selective ligand‐binding activities of these compounds were successfully detected in this yeast RGA. RGA using DmEcR‐DmUSP was the most responsive to the THQ compound, while RGA with CsEcR‐CsUSP was highly responsive to DBHs (Fig. 5 and Table 2). This result is consistent with previous findings showing that dipteran EcR–USP specifically responds to THQ compounds 40, 48, while lepidopteran EcR–USP strongly binds to DBHs 1, 49. Previous studies suggested that the insect order‐selective effects of nonsteroidal ecdysone agonists were due to differences in ligand–receptor‐binding affinity rather than pharmacokinetic and metabolic differences 73, 74. The molecular phylogenetic tree based on the EcR sequences is significantly correlated with the taxonomic classification of various insect species with considerable divergences in the ligand‐binding domain (LBD) among the orders 22, 75, 76. It has been elucidated that DBHs have high accessibility to the ligand‐binding cavity of the lepidopteran EcR–USP compared with other insect orders' EcR–USPs, while the structures of EcR/ecdysteroids complexes are similar among various orders, which may be a molecular basis for the taxonomic order selectivity of DBHs 2, 20, 77, 78, 79, 80, 81, 82. The mutational analysis and the quantitative structure‐activity relationship (QSAR) study suggested that the binding sites of the THQ compounds within the LBD of EcR differ from those of natural ecdysteroids and DBHs, and the THQ‐bound EcR–USP may take the taxonomic order/species‐selective active conformation 23, 27, 41, 83. These findings explain why nonsteroidal ecdysone agonists show insect order‐selective affinity against EcR–USP, while natural ecdysteroids bind to EcR with similar affinity among insect orders.

As shown in Fig. 6, activity in terms of the pEC_50_ of DBHs assessed in the yeast RGA linearly correlated with ligand‐binding activity (pIC_50_) measured using *in vitro* translated EcR–USP 49. However, the activity (pEC_50_) of ecdysteroids measured in the yeast RGA was ~ 1/100 that of the binding affinity (pIC_50_; Fig. 6). This may have been due to the poor uptake of ecdysteroids by yeast cells with cell walls. The deletion of genes encoding cell wall mannoproteins (*CWP1/CWP2*) and/or plasma membrane efflux pumps (*PDR5/PDR10*) may effectively alter the permeability of cell walls and increase intracellular ligand concentrations 36, 84, 85, 86. Deletion of the *ERG6* gene, which is involved in the synthesis of ergosterol, may also effectively enhance membrane permeability 87, 88. Synthetic nonsteroidal ecdysone agonists such as DBHs and THQs may be highly permeable to yeast cells, unlike natural ecdysteroids, and this may be due to the higher hydrophobicity of nonsteroids 11.

The receptor‐binding activities of nonsteroidal ecdysone agonists were previously reported to strongly correlate with the molting hormone activity, toxicity, and transactivation activity of EcR–USP in cultured insect cells 12, 23, 89, 90, 91, 92. Therefore, we may be able to clarify whether nonsteroidal test compounds possess hormonal or insecticidal activity in insects using this yeast RGA.

Insect growth regulators such as ecdysone agonists and juvenile hormone analogs are considered to be safe for mammals and ideal to control insect pests because these chemicals disrupt insect‐specific hormonal events 1. Some DBHs have been developed as insecticides 2, 93. Since genes encoding EcR and USP have been identified in a wide variety of insects 19, we can establish insect species‐specific RGA to detect ecdysone agonists.

Our yeast RGAs for nuclear receptor ligands can be carried out with quite simple and easy procedures, which guarantee robustness of these systems [29, 31, 32, 33, 35, 36, 37]. Our yeast RGAs for the vertebrate nuclear receptors found ligand activity in the environmental samples and human body wastes 30, 32, 34, 36, 37, and detected agonistic and antagonistic activities of various ligands 33, 36, which indicate that our newly established yeast RGAs for the EcR–USP of *D. melanogaster*,* C. suppressalis*, and *L. decemlineata* may be valuable as a screening tool for novel ecdysone agonists and antagonist in plant extracts or large chemical libraries, and for synthetic IGR contamination in the environment. The RGAs will be further developed as a high throughput screening system for the EcR ligands.

## Author contributions

TY designed the research project and supported financially. MM and SIH performed most experiments and analyzed data. YN provided the THQ compound and materials for plasmid construction. MK and YN made comments on the experiments throughout the study. SIH, YN, and TY prepared the manuscript. All authors approved the final version of the manuscript.

## Supporting information


**Fig. S1.** Optimization of RGAs for insect EcR–USPs.

**Fig. S2.** No cross‐reactivities of EcR–USP assay yeasts against vertebrate steroid hormones.

**Fig. S3.** No responses of EcR–USP assay yeasts against alkylphenol compounds.

**Table S1.** Primer sequences.
**Table S2.** Reporter plasmids used in this study.

## References

[1] Dhadialla TS , Carlson GR and Le DP (1998) New insecticides with ecdysteroidal and juvenile hormone activity. Annu Rev Entomol 43, 545–569. 9444757 10.1146/annurev.ento.43.1.545

[2] Dinan L , Nakagawa Y and Hormann RE (2012) Structure‐activity relationships of ecdysteroids and non‐steroidal ecdysone agonists. Adv Insect Physiol 43, 251–298.

[3] Becker E (1941) Über Versuche zur Anreicherung und physiologischer Charakterisierung des Wirkstoffes der Puparisierung. Biol Zbl 61, 360–388.

[4] Sato Y , Sakai M and Fujioka S (1968) Ecdysone activity of plant‐originated molting hormones applied on the body surface of lepidopterous larvae. Appl Entomol Zool 3, 49–51.

[5] Frisrom JW and Yund MA (1976) Characteristics of the action of ecdysones on *Drosophila* imaginal discs cultured in vitro In Invertebrate Tissue Culture Research Application (Maramorosch K, ed.), pp. 161–178. Academic Press, New York.

[6] Nishioka T , Fujita T and Nakajima M (1979) Effect on chitin synthesis inhibitors on cuticle formation of the cultured integument of *Chilo suppressalis* . J Pestic Sci 4, 367–374.

[7] Imoto S , Nishioka T , Fujita T and Nakajima M (1982) Hormonal requirements for the larval‐pupal ecdysis induced in the cultured integument of *Chilo suppressalis* . J Insect Physiol 28, 1025–1033.

[8] Oikawa N , Nakagawa Y , Soya Y , Nishimura K , Kurihara N , Ueno T and Fujita T (1993) Enhancement of N‐acetylglucosamine incorporation into the cultured integument of *Chilo suppressalis* by molting hormone and dibenzoylhydrazine insecticides. Pestic Biochem Physiol 47, 165–170.

[9] Nakagawa Y , Nishimura K , Oikawa N , Kurihara N and Ueno T (1995) Activity of ecdysone analogs in enhancing N‐acetylglucosamine incorporation into the cultured integument of *Chilo suppressalis* . Steroids 60, 401–405. 7570713 10.1016/0039-128x(94)00065-k

[10] Nakagawa Y , Minakuchi C and Ueno T (2000) Inhibition of [(3)H]ponasterone a binding by ecdysone agonists in the intact Sf‐9 cell line. Steroids 65, 537–542. 10978733 10.1016/s0039-128x(00)00130-6

[11] Nakagawa Y , Minakuchi C , Takahashi K and Ueno T (2002) Inhibition of [3H]ponasterone A binding by ecdysone agonists in the intact Kc cell line. Insect Biochem Mol Biol 32, 175–180. 11755060 10.1016/s0965-1748(01)00105-9

[12] Minakuchi C , Nakagawa Y , Kamimura M and Miyagawa H (2003) Binding affinity of nonsteroidal ecdysone agonists against the ecdysone receptor complex determines the strength of their molting hormonal activity. Eur J Biochem 270, 4095–4104. 14519121 10.1046/j.1432-1033.2003.03801.x

[13] Ogura T , Nakagawa Y , Minakuchi C and Miyagawa H (2005) QSAR for binding affinity of substituted dibenzoylhydrazines to intact Sf‐9 cells. J Pestic Sci 30, 1–6.

[14] Karlson P and Shaaya E (1964) Der Ecdysontiter Wahrend Der Insektenentwicklung. 1. Eine Methode Zur Bestimmung Des Ecdysongehalts. J Insect Physiol 10, 797–804.

[15] Huber R and Hoppe W (1965) Zur Chemie des Ecdysons, VII: Die Kristall‐ und Molekulstructuranalyse des Insektenverpuppungshormons Ecdyson mit der automatisierten Faltmolekulmethode. Chem Ber 98, 2403–2424. 5849856 10.1002/cber.19650980744

[16] Koelle MR , Talbot WS , Segraves WA , Bender MT , Cherbas P and Hogness DS (1991) The *Drosophila* EcR gene encodes an ecdysone receptor, a new member of the steroid receptor superfamily. Cell 67, 59–77. 1913820 10.1016/0092-8674(91)90572-g

[17] Yao TP , Segraves WA , Oro AE , McKeown M and Evans RM (1992) *Drosophila* ultraspiracle modulates ecdysone receptor function via heterodimer formation. Cell 71, 63–72. 1327536 10.1016/0092-8674(92)90266-f

[18] Yao TP , Forman BM , Jiang Z , Cherbas L , Chen JD , McKeown M , Cherbas P and Evans RM (1993) Functional ecdysone receptor is the product of EcR and Ultraspiracle genes. Nature 366, 476–479. 8247157 10.1038/366476a0

[19] Nakagawa Y and Henrich VC (2009) Arthropod nuclear receptors and their role in molting. FEBS J 276, 6128–6157. 19796154 10.1111/j.1742-4658.2009.07347.x

[20] Billas IM , Iwema T , Garnier JM , Mitschler A , Rochel N and Moras D (2003) Structural adaptability in the ligand‐binding pocket of the ecdysone hormone receptor. Nature 426, 91–96. 14595375 10.1038/nature02112

[21] Harada T , Nakagawa Y , Ogura T , Yamada Y , Ohe T and Miyagawa H (2011) Virtual screening for ligands of the insect molting hormone receptor. J Chem Inf Model 51, 296–305. 21275397 10.1021/ci100400k

[22] Ogura T , Minakuchi C , Nakagawa Y , Smagghe G and Miyagawa H (2005) Molecular cloning, expression analysis and functional confirmation of ecdysone receptor and ultraspiracle from the Colorado potato beetle *Leptinotarsa decemlineata* . FEBS J 272, 4114–4128. 16098194 10.1111/j.1742-4658.2005.04823.x

[23] Soin T , Swevers L , Kotzia G , Iatrou K , Janssen CR , Rouge P , Harada T , Nakagawa Y and Smagghe G (2010) Comparison of the activity of non‐steroidal ecdysone agonists between dipteran and lepidopteran insects, using cell‐based EcR reporter assays. Pest Manag Sci 66, 1215–1229. 20672340 10.1002/ps.1998

[24] Mikitani K (1995) Sensitive, rapid and simple method for evaluation of ecdysteroid agonist activity based on the mode of action of the hormone. J Seric Sci Jpn 64, 534–539.

[25] Tanaka K , Tsukamoto Y , Sawada Y , Kasuya A , Hotta H , Ichinose R , Watanabe T , Toya T , Yokoi S , Kawagishi A *et al* (2002) Chromafenozide: a novel lepidopteran insect control agent. Ann Rep Sankyo Res Lab 53, 1–28.

[26] Dai X , Willis LG , Palli SR and Theilmann DA (2005) Tight transcriptional regulation of foreign genes in insect cells using an ecdysone receptor‐based inducible system. Protein Expr Purif 42, 236–245. 15936954 10.1016/j.pep.2004.12.031

[27] Soin T , De Geyter E , Mosallanejad H , Iga M , Martin D , Ozaki S , Kitsuda S , Harada T , Miyagawa H , Stefanou D *et al* (2010) Assessment of species specificity of moulting accelerating compounds in Lepidoptera: comparison of activity between *Bombyx mori* and *Spodoptera littoralis* by in vitro reporter and in vivo toxicity assays. Pest Manag Sci 66, 526–535. 20069627 10.1002/ps.1903

[28] Ogura T , Nakagawa Y , Swevers L , Smagghe G and Miyagawa H (2012) Quantitative evaluation of the molting hormone activity in coleopteran cells established from the Colorado potato beetle, *Leptinotarsa decemlineata* . Pestic Biochem Physiol 104, 1–8.

[29] Kawanishi M , Sakamoto M , Ito A , Kishi K and Yagi T (2003) Construction of reporter yeasts for mouse aryl hydrocarbon receptor ligand activity. Mutat Res 540, 99–105. 12972062 10.1016/s1383-5718(03)00174-8

[30] Kawanishi M , Takamura‐Enya T , Ermawati R , Shimohara C , Sakamoto M , Matsukawa K , Matsuda T , Murahashi T , Matsui S , Wakabayashi K *et al* (2004) Detection of genistein as an estrogenic contaminant of river water in Osaka. Environ Sci Technol 38, 6424–6429. 15597900 10.1021/es049764v

[31] Kawanishi M , Sakamoto M , Shimohara C and Yagi T (2006) Establishment of reporter yeasts for guinea pig and syrian hamster aryl hydrocarbon receptor ligand activity. Genes Environ 28, 167–172.

[32] Chu WL , Shiizaki K , Kawanishi M , Kondo M and Yagi T (2009) Validation of a new yeast‐based reporter assay consisting of human estrogen receptors alpha/beta and coactivator SRC‐1: application for detection of estrogenic activity in environmental samples. Environ Toxicol 24, 513–521. 19161236 10.1002/tox.20473

[33] Shiizaki K , Asai S , Ebata S , Kawanishi M and Yagi T (2010) Establishment of yeast reporter assay systems to detect ligands of thyroid hormone receptors alpha and beta. Toxicol In Vitro 24, 638–644. 19853653 10.1016/j.tiv.2009.10.001

[34] Kawanishi M , Ohnisi K , Takigami H and Yagi T (2013) Simple and rapid yeast reporter bioassay for dioxin screening: evaluation of the dioxin‐like compounds in industrial and municipal waste incineration plants. Environ Sci Pollut Res Int 20, 2993–3002. 23054780 10.1007/s11356-012-1214-4

[35] Shiizaki K , Yoshikawa T , Takada E , Hirose S , Ito‐Harashima S , Kawanishi M and Yagi T (2014) Development of yeast reporter assay for screening specific ligands of retinoic acid and retinoid X receptor subtypes. J Pharmacol Toxicol Methods 69, 245–252. 24530888 10.1016/j.vascn.2014.01.007

[36] Ito‐Harashima S , Shiizaki K , Kawanishi M , Kakiuchi K , Onishi K , Yamaji R and Yagi T (2015) Construction of sensitive reporter assay yeasts for comprehensive detection of ligand activities of human corticosteroid receptors through inactivation of CWP and PDR genes. J Pharmacol Toxicol Methods 74, 41–52. 26070404 10.1016/j.vascn.2015.06.001

[37] Matsui S , Ito‐Harashima S , Sugimoto Y , Takada E , Shiizaki K , Kawanishi M and Yagi T (2016) Development of yeast reporter assays for the enhanced detection of environmental ligands of thyroid hormone receptors alpha and beta from *Xenopus tropicalis* . Toxicol In Vitro 37, 15–24. 27544454 10.1016/j.tiv.2016.08.008

[38] Bai J , Uehara Y and Montell DJ (2000) Regulation of invasive cell behavior by taiman, a *Drosophila* protein related to AIB1, a steroid receptor coactivator amplified in breast cancer. Cell 103, 1047–1058. 11163181 10.1016/s0092-8674(00)00208-7

[39] Burke D , Dawson D and Stearns T (2000) Methods in Yeast Genetics – A Laboratory Course Manual, Cold Spring Harbor (2000 edition). Cold Spring Harbor Laboratory Press, Cold Spring Harbor, NY.

[40] Smith HC , Cavanaugh CK , Friz JL , Thompson CS , Saggers JA , Michelotti EL , Garcia J and Tice CM (2003) Synthesis and SAR of cis‐1‐benzoyl‐1,2,3,4‐tetrahydroquinoline ligands for control of gene expression in ecdysone responsive systems. Bioorg Med Chem Lett 13, 1943–1946. 12749904 10.1016/s0960-894x(03)00317-2

[41] Kitamura S , Harada T , Hiramatsu H , Shimizu R , Miyagawa H and Nakagawa Y (2014) Structural requirement and stereospecificity of tetrahydroquinolines as potent ecdysone agonists. Bioorg Med Chem Lett 24, 1715–1718. 24630413 10.1016/j.bmcl.2014.02.043

[42] Hu X , Cherbas L and Cherbas P (2003) Transcription activation by the ecdysone receptor (EcR/USP): identification of activation functions. Mol Endocrinol 17, 716–731. 12554759 10.1210/me.2002-0287

[43] Minakuchi C , Nakagawa Y , Soya Y and Miyagawa H (2004) Preparation of functional ecdysteroid receptor proteins (EcR and USP) using a wheat germ cell‐free protein synthesis system. J Pestic Sci 29, 189–194.

[44] Wolf SS , Roder K and Schweizer M (1996) Construction of a reporter plasmid that allows expression libraries to be exploited for the one‐hybrid system. Biotechniques 20, 568–574. 8800671 10.2144/19962004568

[45] Gietz RD , Schiestl RH , Willems AR and Woods RA (1995) Studies on the transformation of intact yeast cells by the LiAc/SS‐ DNA/PEG procedure. Yeast 11, 355–360. 7785336 10.1002/yea.320110408

[46] Sakuma M (1998) Probit analysis of preference data. Appl Entomol Zool 33, 339–347.

[47] Riddihough G and Pelham HR (1987) An ecdysone response element in the *Drosophila* hsp27 promoter. EMBO J 6, 3729–3734. 16453813 10.1002/j.1460-2075.1987.tb02707.x PMC553843

[48] Palli SR , Tice CM , Margam VM and Clark AM (2005) Biochemical mode of action and differential activity of new ecdysone agonists against mosquitoes and moths. Arch Insect Biochem Physiol 58, 234–242. 15756700 10.1002/arch.20046

[49] Minakuchi C , Ogura T , Miyagawa H and Nakagawa Y (2007) Effects of the structures of ecdysone receptor (EcR) and ultraspiracle (USP) on the ligand‐binding activity of the EcR/USP heterodimer. J Pestic Sci 32, 379–384.

[50] Talbot WS , Swyryd EA and Hogness DS (1993) *Drosophila* tissues with different metamorphic responses to ecdysone express different ecdysone receptor isoforms. Cell 73, 1323–1337. 8324824 10.1016/0092-8674(93)90359-x

[51] Thomas HE , Stunnenberg HG and Stewart AF (1993) Heterodimerization of the *Drosophila* ecdysone receptor with retinoid X receptor and ultraspiracle. Nature 362, 471–475. 8385270 10.1038/362471a0

[52] Palli SR , Kapitskaya MZ , Kumar MB and Cress DE (2003) Improved ecdysone receptor‐based inducible gene regulation system. Eur J Biochem 270, 1308–1315. 12631289 10.1046/j.1432-1033.2003.03501.x

[53] Tran HT , Askari HB , Shaaban S , Price L , Palli SR , Dhadialla TS , Carlson GR and Butt TR (2001) Reconstruction of ligand‐dependent transactivation of *Choristoneura fumiferana* ecdysone receptor in yeast. Mol Endocrinol 15, 1140–1153. 11435614 10.1210/mend.15.7.0660

[54] Tran HT , Shaaban S , Askari HB , Walfish PG , Raikhel AS and Butt TR (2001) Requirement of co‐factors for the ligand‐mediated activity of the insect ecdysteroid receptor in yeast. J Mol Endocrinol 27, 191–209. 11564603 10.1677/jme.0.0270191

[55] Dela Cruz F and Mak P (1997) *Drosophila* ecdysone receptor functions as a constitutive activator in yeast. J Steroid Biochem Mol Biol 62, 353–359. 9408090 10.1016/s0960-0760(97)00046-0

[56] Dela Cruz FE , Kirsch DR and Heinrich JN (2000) Transcriptional activity of *Drosophila melanogaster* ecdysone receptor isoforms and ultraspiracle in *Saccharomyces cerevisiae* . J Mol Endocrinol 24, 183–191. 10750019 10.1677/jme.0.0240183

[57] Lezzi M , Bergman T , Henrich VC , Vogtli M , Fromel C , Grebe M , Przibilla S and Spindler‐Barth M (2002) Ligand‐induced heterodimerization between the ligand binding domains of the *Drosophila* ecdysteroid receptor and ultraspiracle. Eur J Biochem 269, 3237–3245. 12084064 10.1046/j.1432-1033.2002.03001.x

[58] Xu J and Li Q (2003) Review of the in vivo functions of the p160 steroid receptor coactivator family. Mol Endocrinol 17, 1681–1692. 12805412 10.1210/me.2003-0116

[59] Horner MA , Chen T and Thummel CS (1995) Ecdysteroid regulation and DNA binding properties of *Drosophila* nuclear hormone receptor superfamily members. Dev Biol 168, 490–502. 7729584 10.1006/dbio.1995.1097

[60] Antoniewski C , Mugat B , Delbac F and Lepesant JA (1996) Direct repeats bind the EcR/USP receptor and mediate ecdysteroid responses in *Drosophila melanogaster* . Mol Cell Biol 16, 2977–2986. 8649409 10.1128/mcb.16.6.2977 PMC231292

[61] Wang SF , Miura K , Miksicek RJ , Segraves WA and Raikhel AS (1998) DNA binding and transactivation characteristics of the mosquito ecdysone receptor‐Ultraspiracle complex. J Biol Chem 273, 27531–27540. 9765285 10.1074/jbc.273.42.27531

[62] Martin D , Wang SF and Raikhel AS (2001) The vitellogenin gene of the mosquito *Aedes aegypti* is a direct target of ecdysteroid receptor. Mol Cell Endocrinol 173, 75–86. 11223179 10.1016/s0303-7207(00)00413-5

[63] Perera SC , Zheng S , Feng QL , Krell PJ , Retnakaran A and Palli SR (2005) Heterodimerization of ecdysone receptor and ultraspiracle on symmetric and asymmetric response elements. Arch Insect Biochem Physiol 60, 55–70. 16175536 10.1002/arch.20081

[64] Minakuchi C , Nakagawa Y , Kiuchi M , Seino A , Tomita S and Kamimura M (2003) Molecular cloning and expression analysis of ultraspiracle (USP) from the rice stem borer *Chilo suppressalis* . Insect Biochem Mol Biol 33, 41–49. 12459199 10.1016/s0965-1748(02)00165-0

[65] Kuiper GG , Lemmen JG , Carlsson B , Corton JC , Safe SH , van der Saag PT , van der Burg B and Gustafsson JA (1998) Interaction of estrogenic chemicals and phytoestrogens with estrogen receptor beta. Endocrinology 139, 4252–4263. 9751507 10.1210/endo.139.10.6216

[66] Dinan L , Bourne P , Whiting P , Dhadialla TS and Hutchinson TH (2001) Screening of environmental contaminants for ecdysteroid agonist and antagonist activity using the *Drosophila melanogaster* B(II) cell in vitro assay. Environ Toxicol Chem 20, 2038–2046. 11521832 10.1897/1551-5028(2001)020<2038:soecfe>2.0.co;2

[67] Kitamura S , Kato T , Iida M , Jinno N , Suzuki T , Ohta S , Fujimoto N , Hanada H , Kashiwagi K and Kashiwagi A (2005) Anti‐thyroid hormonal activity of tetrabromobisphenol A, a flame retardant, and related compounds: affinity to the mammalian thyroid hormone receptor, and effect on tadpole metamorphosis. Life Sci 76, 1589–1601. 15680168 10.1016/j.lfs.2004.08.030

[68] Kudo Y and Yamauchi K (2005) In vitro and in vivo analysis of the thyroid disrupting activities of phenolic and phenol compounds in *Xenopus laevis* . Toxicol Sci 84, 29–37. 15590892 10.1093/toxsci/kfi049

[69] Bonefeld‐Jorgensen EC , Long M , Hofmeister MV and Vinggaard AM (2007) Endocrine‐disrupting potential of bisphenol A, bisphenol A dimethacrylate, 4‐n‐nonylphenol, and 4‐n‐octylphenol in vitro: new data and a brief review. Environ Health Perspect 115 (Suppl 1), 69–76. 18174953 10.1289/ehp.9368 PMC2174402

[70] Svoboda JA , Kaplanis JN , Robbins WE and Thompson MJ (1975) Recent developments in insect steroid metabolism. Annu Rev Entomol 20, 205–220. 1091201 10.1146/annurev.en.20.010175.001225

[71] Clement CY , Bradbrook DA , Lafont R and Dinan L (1993) Assessment of a microplate‐based bioassay for the detection of ecdysteroid‐like or antiecdysteroid activities. Insect Biochem Mol Biol 23, 187–193.

[72] Harmatha J and Dinan L (1997) Biological activity of natural and synthetic ecdysteroids in the BII bioassay. Arch Insect Biochem Physiol 35, 219–225. 9131786 10.1002/(SICI)1520-6327(1997)35:1/2<219::AID-ARCH20>3.0.CO;2-D

[73] Smagghe G and Degheele D (1993) Metabolism, pharmacokinetics, and toxicity of the first nonsteroidal ecdysteroid agonist RH 5849 to *Spodoptera exempta* (Walker), *Spodoptera exigua* (Hübner), and *Leptinotarsa decemlineata* (Say). Pestic Biochem Physiol 46, 149–160.

[74] Smagghe G and Degheele D (1994) The significance of pharmacokinetics and metabolism to the biological activity of RH‐5992 (Tebufenozide) in *Spodoptera exempta*,* Spodoptera exigua*, and *Leptinotarsa decemlineata* . Pestic Biochem Physiol 49, 224–234.

[75] Bonneton FO , Zelus D , Iwema T , Robinson‐Rechavi M and Laudet V (2003) Rapid divergence of the ecdysone receptor in Diptera and Lepidoptera suggests coevolution between ECR and USP‐RXR. Mol Biol Evol 20, 541–553. 12654933 10.1093/molbev/msg054

[76] Nakagawa Y , Sakai A , Magata F , Ogura T , Miyashita M and Miyagawa H (2007) Molecular cloning of the ecdysone receptor and the retinoid X receptor from the scorpion *Liocheles australasiae* . FEBS J 274, 6191–6203. 18028192 10.1111/j.1742-4658.2007.06139.x

[77] Kumar MB , Fujimoto T , Potter DW , Deng Q and Palli SR (2002) A single point mutation in ecdysone receptor leads to increased ligand specificity: implications for gene switch applications. Proc Natl Acad Sci USA 99, 14710–14715. 12411578 10.1073/pnas.222278999 PMC137484

[78] Carmichael JA , Lawrence MC , Graham LD , Pilling PA , Epa VC , Noyce L , Lovrecz G , Winkler DA , Pawlak‐Skrzecz A , Eaton RE *et al* (2005) The X‐ray structure of a hemipteran ecdysone receptor ligand‐binding Domain: comparison with a Lepidopteran ecdysone receptor ligand‐binding domain and implications for insecticide design. J Biol Chem 280, 22258–22269. 15809296 10.1074/jbc.M500661200

[79] Hormann RE , Smagghe G and Nakagawa Y (2008) Multidimensional quantitative structure‐activity relationships of diacylhydrazine toxicity to lepidopteran and coleopteran insect pests. QSAR Comb Sci 27, 1098–1112.

[80] Nakagawa Y , Hormann RE and Smagghe G (2009) SAR and QSAR studies for in vivo and in vitro activities of ecdysone agonists In Ecdysone: Structures and Functions (Smagghe G, ed.), pp. 475–509. Springer Netherlands, Dordrecht.

[81] Morou E , Lirakis M , Pavlidi N , Zotti M , Nakagawa Y , Smagghe G , Vontas J and Swevers L (2013) A new dibenzoylhydrazine with insecticidal activity against Anopheles mosquito larvae. Pest Manag Sci 69, 827–833. 23208761 10.1002/ps.3441

[82] Zotti MJ , De Geyter E , Swevers L , Braz ASK , Scott LPB , Rougé P , Coll J , Grutzmacher AD , Lenardão EJ and Smagghe G (2013) A cell‐based reporter assay for screening for EcR agonist/antagonist activity of natural ecdysteroids in Lepidoptera (Bm5) and Diptera (S2) cell cultures, followed by modeling of ecdysteroid‐EcR interactions and normal mode analysis. Pestic Biochem Physiol 107, 309–320. 24267692 10.1016/j.pestbp.2013.09.003

[83] Kumar MB , Potter DW , Hormann RE , Edwards A , Tice CM , Smith HC , Dipietro MA , Polley M , Lawless M , Wolohan PR *et al* (2004) Highly flexible ligand binding pocket of ecdysone receptor: a single amino acid change leads to discrimination between two groups of nonsteroidal ecdysone agonists. J Biol Chem 279, 27211–27218. 15107428 10.1074/jbc.M403839200

[84] Kralli A , Bohen SP and Yamamoto KR (1995) LEM1, an ATP‐binding‐cassette transporter, selectively modulates the biological potency of steroid hormones. Proc Natl Acad Sci USA 92, 4701–4705. 7753868 10.1073/pnas.92.10.4701 PMC42012

[85] Zhang M , Liang Y , Zhang X , Xu Y , Dai H and Xiao W (2008) Deletion of yeast CWP genes enhances cell permeability to genotoxic agents. Toxicol Sci 103, 68–76. 18281714 10.1093/toxsci/kfn034

[86] Zhang M , Hanna M , Li J , Butcher S , Dai H and Xiao W (2010) Creation of a hyperpermeable yeast strain to genotoxic agents through combined inactivation of PDR and CWP genes. Toxicol Sci 113, 401–411. 19884123 10.1093/toxsci/kfp267

[87] Sitcheran R , Emter R , Kralli A and Yamamoto KR (2000) A genetic analysis of glucocorticoid receptor signaling. Identification and characterization of ligand‐effect modulators in *Saccharomyces cerevisiae* . Genetics 156, 963–972. 11063677 10.1093/genetics/156.3.963 PMC1461341

[88] Emter R , Heese‐Peck A and Kralli A (2002) ERG6 and PDR5 regulate small lipophilic drug accumulation in yeast cells via distinct mechanisms. FEBS Lett 521, 57–61. 12067726 10.1016/s0014-5793(02)02818-1

[89] Smagghe G , Eelen H , Verschelde E , Richter K and Degheele D (1996) Differential effects of nonsteroidal ecdysteroid agonists in coleoptera and lepidoptera: analysis of evagination and receptor binding in imaginal discs. Insect Biochem Mol Biol 26, 687–695.

[90] Nakagawa Y , Hattori K , Minakuchi C , Kugimiya S and Ueno T (2000) Relationships between structure and molting hormonal activity of tebufenozide, methoxyfenozide, and their analogs in cultured integument system of *Chilo suppressalis* Walker. Steroids 65, 117–123. 10699589 10.1016/s0039-128x(99)00091-4

[91] Carlson GR , Dhadialla TS , Hunter R , Jansson RK , Jany CS , Lidert Z and Slawecki RA (2001) The chemical and biological properties of methoxyfenozide, a new insecticidal ecdysteroid agonist. Pest Manag Sci 57, 115–119. 11455641 10.1002/1526-4998(200102)57:2<115::AID-PS245>3.0.CO;2-A

[92] Smagghe G , Dhadialla TS and Lezzi M (2002) Comparative toxicity and ecdysone receptor affinity of non‐steroidal ecdysone agonists and 20‐hydroxyecdysone in *Chironomus tentans* . Insect Biochem Mol Biol 32, 187–192. 11755062 10.1016/s0965-1748(01)00109-6

[93] Nakagawa Y (2005) Nonsteroidal ecdysone agonists. Vitam Horm 73, 131–173. 16399410 10.1016/S0083-6729(05)73005-3

