# Supplementary figures and images for: New reporter gene assays for detecting natural and synthetic molting hormone agonists using yeasts expressing ecdysone receptors of various insects
Source: FEBS Open Bio. 2017 Jun 5;7(7):995–1008. doi: 10.1002/2211-5463.12239 (PMC5494300; doi:10.1002/2211-5463.12239)

Fig. S1

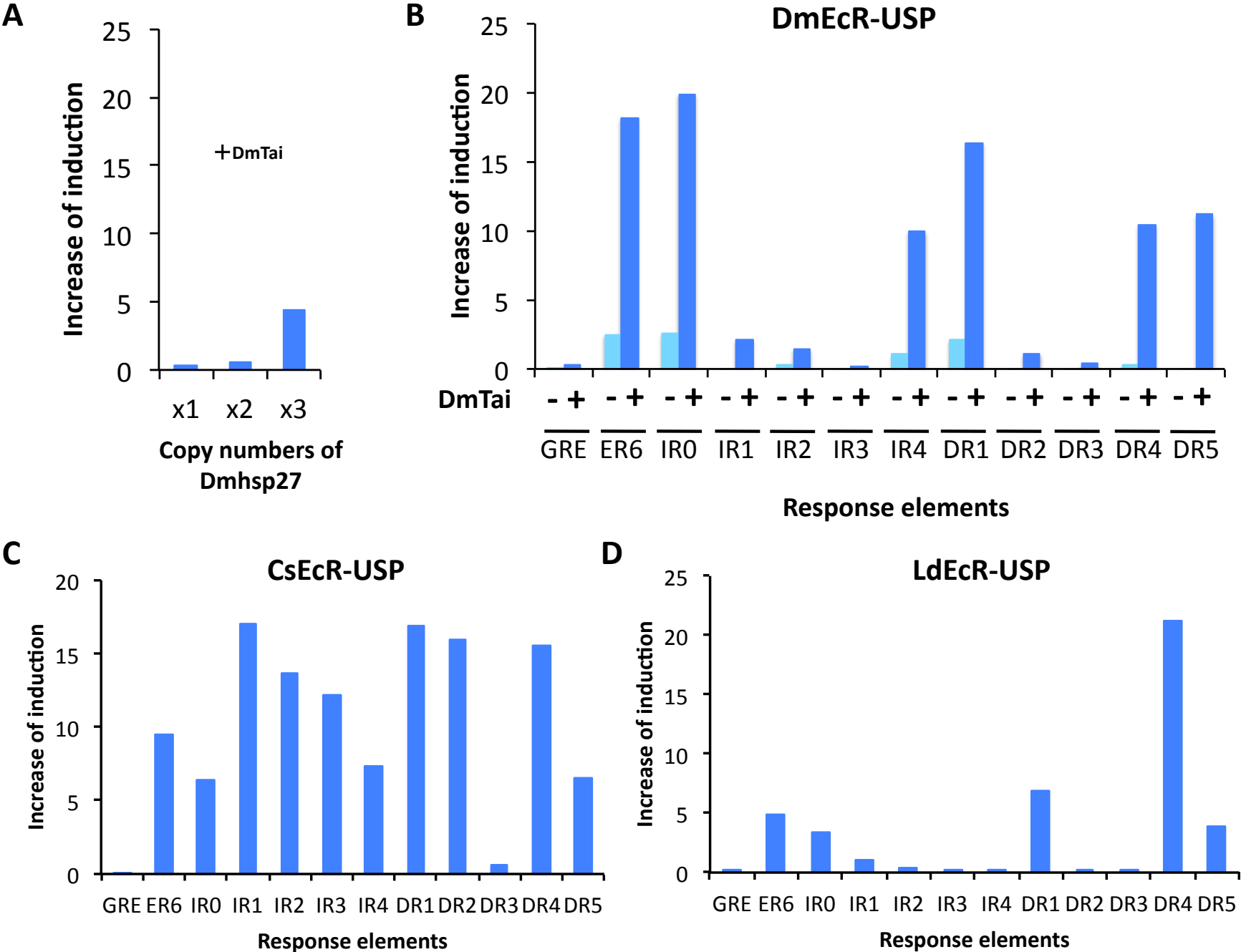

Supplement: Supplementary file 1 — Fig. S1. Optimization of RGAs for insect EcR–USPs. [file FEB4-7-995-s001.pdf]

Fig. S2

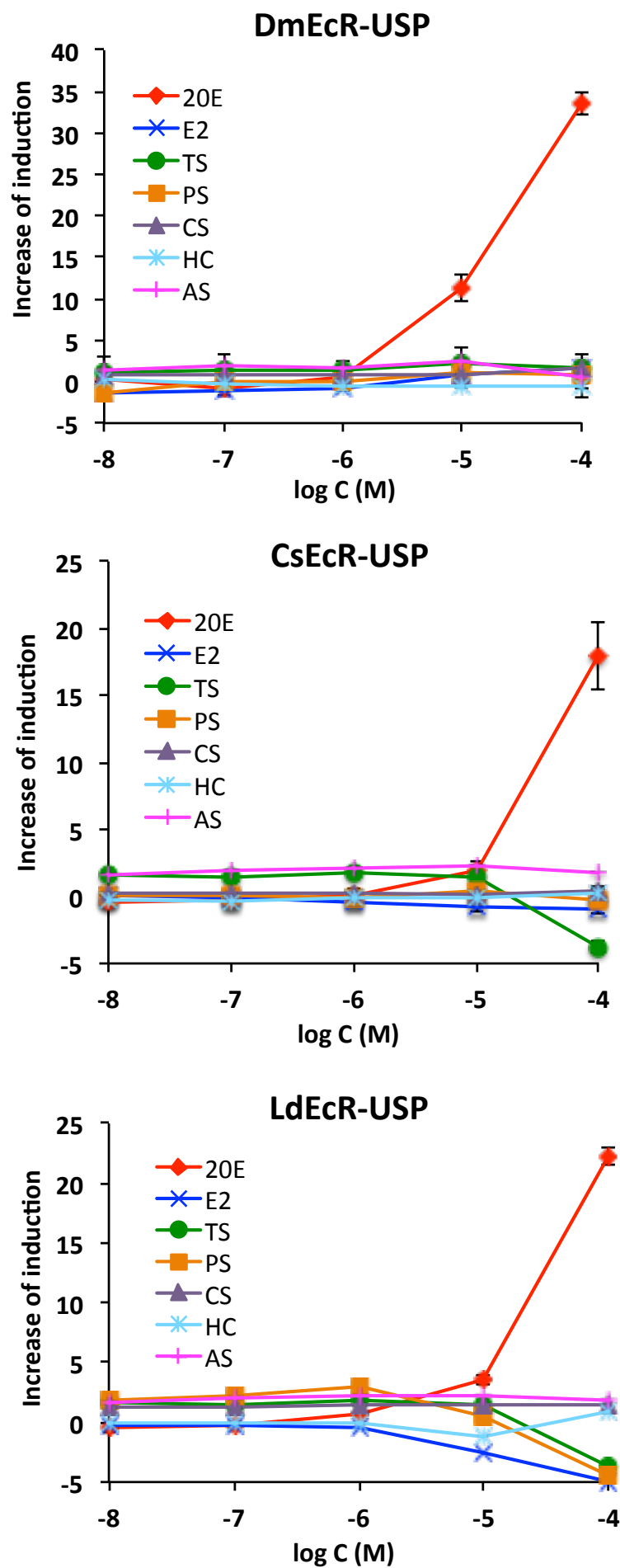

Supplement: Supplementary file 2 — Fig. S2. No cross‐reactivities of EcR–USP assay yeasts against vertebrate steroid hormones. [file FEB4-7-995-s002.pdf]

Fig. S3

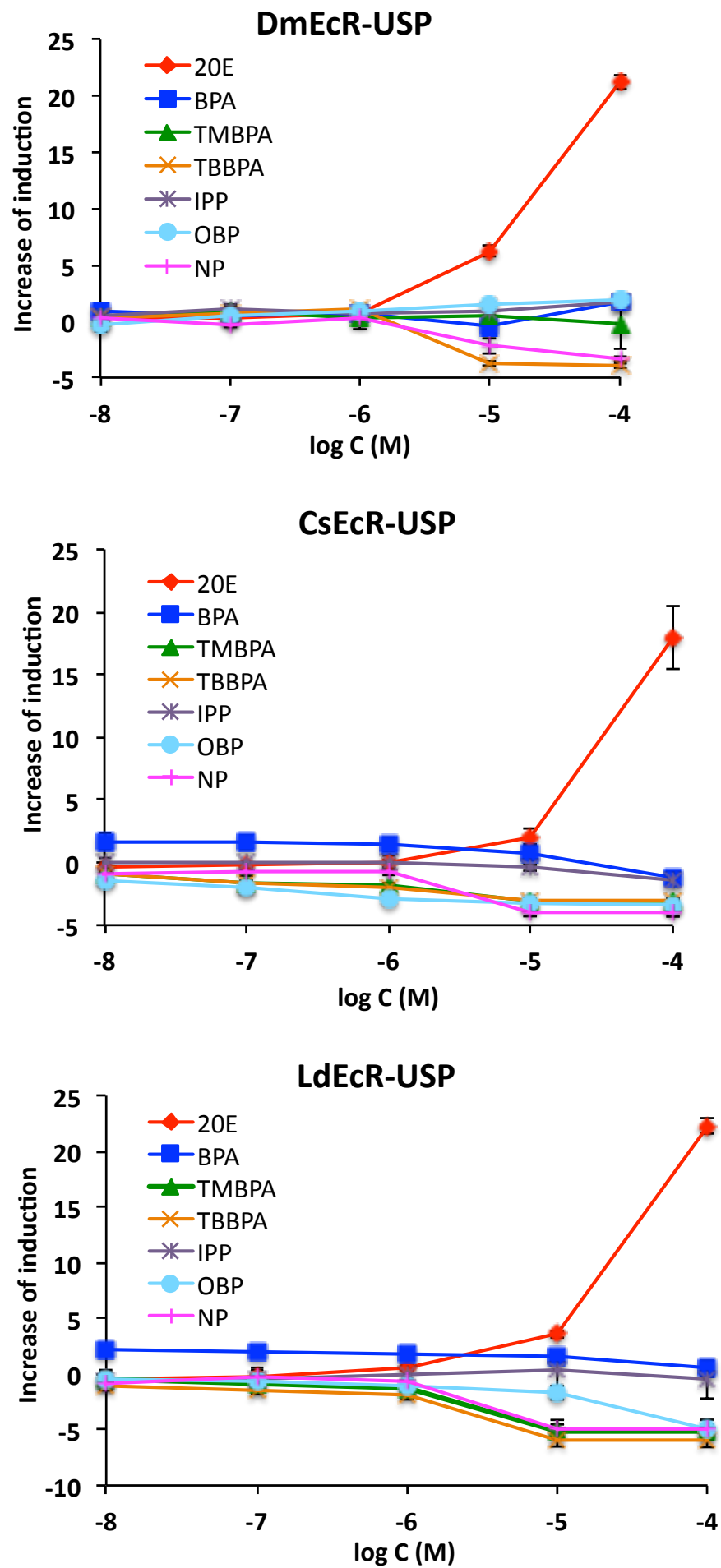

Supplement: Supplementary file 3 — Fig. S3. No responses of EcR–USP assay yeasts against alkylphenol compounds. [file FEB4-7-995-s003.pdf]
