# Supplementary material for: New reporter gene assays for detecting natural and synthetic molting hormone agonists using yeasts expressing ecdysone receptors of various insects
Source: FEBS Open Bio. 2017 Jun 5;7(7):995–1008. doi: 10.1002/2211-5463.12239 (PMC5494300; doi:10.1002/2211-5463.12239)
Supplement: Supplementary file 4 — Table S1. Primer sequences. Table S2. Reporter plasmids used in this study. [file FEB4-7-995-s004.docx]

**Table S-1. Primer sequences**

| Primer | Sequence (5’-3’) | Comments |
| --- | --- | --- |
| DmEcR-Fwd^a, b^ | CCCCGTCGAC*AACAAA*ATGAAGCGCGCTGGTCGAA | Amplify DmEcR ORF |
| DmEcR-Rev^a^ | CCCCAAGCTTCTATGCAGTCGTCGAGTGCTC | Amplify DmEcR ORF |
| DmUSP-Fwd^a, b^ | CCCCCCCGGG*AACAAA*ATGGACAACTGCGACCAGGAC | Amplify DmUSP ORF |
| DmUSP-Rev^a^ | CCCCGAATTCCTACTCCAGTTTCATCGCCAG | Amplify DmUSP ORF |
| CsEcR-Fwd^a, b^ | CCCCGGATCC*AACAAA*ATGAGACGCCGTTGGTCCAAC | Amplify CsEcR ORF |
| CsEcR-Rev^a^ | CCCCAAGCTTCTAGAGGTTGGTCGCCACGTC | Amplify CsEcR ORF |
| CsUSP-Fwd^a, b^ | CCCCCCCGGG*AACAAA*ATGGAGCCCTCGAGAGAGCC | Amplify CsUSP ORF |
| CsUSP-Rev^a^ | CCCCGAATTCTTACATTAGAGCAGACGCGTCG | Amplify CsUSP ORF |
| LdEcR-Fwd^a, b^ | CCCCTCTAGA*AACAAA*ATGACCACCATACACTCGATCACC | Amplify LdEcR ORF |
| LdEcR-Rev^a^ | CCCCAAGCTTCTATGTCTTCATGTCGACGTCC | Amplify LdEcR ORF |
| LdUSP-Fwd^a, b^ | CCCCCCCGGG*AACAAA*ATGGGTCCGTTGGGTCCCCAGTCG | Amplify LdUSP ORF |
| LdUSP-Rev^a^ | CCCCGAGCTCCTAAGTATCCGACTGGTTTTCC | Amplify LdUSP ORF |
| DmTai Fwd^a, b^ | CCCCAGATCT*AACAAA*ATGTCAATTGCTGCAGCCGAAAATGCAGG | Amplify DmTai ORF |
| DmTai Rev^a^ | CCCCAAGCTTCTAGAAGCTGAAGCTCAGCGTTGGGTCCGG | Amplify DmTai ORF |
| Dmhsp27 Fwd^c^ | CTAGTCATCGACAAGGGTTCAATGCACTTGTCT | Response element |
| Dmhsp27 Rev^c^ | CTAGAGACAAGTGCATTGAACCCTTGTCGATCA | Response element |

^a^Underlined sequences are additional nucleotides to introduce restriction enzyme recognition sites.

^b^The sequences shown in italics are yeast ribosomal-binding consensus sequences.

^c^ EcRE sequences were underlined.

**Table S-2. Reporter plasmids used in this study.**

| Plasmid name | Sequence of the response element | References |
| --- | --- | --- |
| pYTβ-IR0 × 5 | AGGTCA TGACCT | [33] |
| pYTβ-IR1 × 4 | AGGTCATTGACCT | Laboratory stock |
| pYTβ-IR2 × 5 | AGGTCAGATGACCT | Laboratory stock |
| pYTβ-IR3 × 5 | AGGTCAATTTGACCT | Laboratory stock |
| pYTβ-IR4 × 5 | AGGTCAGAAGTGACCT | Laboratory stock |
| pYTβ-DR1 × 5 | AGGTCAGAGGTCA | [35] |
| pYTβ-DR2 × 4 | AGCGGATAAGGTCA | Laboratory stock |
| pYTβ-DR3 × 2 | AGGTCATTTAGGTCA | Laboratory stock |
| pYTβ-DR4 × 7 | AGGTCAGGAAAGGTCA | [33] |
| pYTβ-DR5 × 5 | AGGTCATTGTAAGGTCA | [35] |
| pYTβ-ER6 × 3 | TGACCTCCTTTGAGGTCA | [33] |
| pYTβ-GRE-R8CS | AGAACAGTTTGTTCT | [36] |
| pYTβ-Dmhsp27 × 1, × 2, × 3 | GACAAGGGTTCAATGCACTTGTC | This study |

* Half sites of the response elements were underlined.
